# Abstract of a Lecture on “How Can Diseases of the Nose and Throat Affect the Teeth?”

**Published:** 1893-09

**Authors:** Edwin Pynchon


### SELECTIONS.

### Abstract of a Lecture on How Can Diseases of the Nose and Throat Affect the Teeth?

BY EDWIN PYNCHON, M.D.

GENTLEMEN:—A study of the diseases of the nose and throat is a thing which I believe has not before been attempted in a dental college. When such a subject is first suggested to the dental student it might seem to him to be somewhat immaterial, though, I am convinced that after investigation, it will be ascertained that, for him, it is of far more importance than is the mastering of the anatomy of the extremities, as this subject deals with the parts in close proximity to his future field of labor, and with parts, a diseased condition of which can, in several ways, affect the growth, development and continued health of the teeth. While general good health tends toward the preservation of the teeth the converse is likewise true, and applies with even more force in case of diseased conditions of adjacent tissues.

In order that a comprehensive understanding of the subject may be acquired it will be necessary to give some particular attention to the anatomy and physiology of the parts under special consideration, though some advantage may be gained in reversing the usual order of these studies by first considering the physiology of the upper air passages, coupled with some general ideas as to their anatomy, and then later on in succeeding lectures while considering the diseases of the different parts, preface each division with a more careful study of the anatomy of the part being considered, so that the normal and the pathological can at every step be compared and correctly understood.

In order to sustain life there are certain necessities which cannot be dispensed with, as food, drink, clothing, sleep and air. A deficiency in any one of these requirements will have an effect upon the health and even upon the life of the individual. Your teacher of physiology will touch upon all these necessities, and I will take the privilege of enlarging somewhat upon the latter and its use in the process of respiration.

The first necessity of the newly-born babe is to breathe the breath of life. In fact, prior to birth, through the medium of the placental circulation, the fœtal blood receives the required oxygenation. Air is demanded much sooner than is either food or drink, and this process of breathing, which is thus begun so early in life, is ceaselessly and constantly continued, whether awake or asleep, sick or well, at work or at play, without intermission or cessation until the light of life has vanished—until the approach to death's cold door is made. In fact, air is our only want which cannot, at times, be dispensed with.

The number of respirations taken per minute is influenced by the age, sex, size and condition of the individual; children breathing much more rapidly than adults, and women more rapidly than men. Fat people respire more rapidly than do spare people, and small people more rapidly than do large people who are not very stout. Generally those whose respirations are rapid inhale less air at each inspiration than do those whose respirations are slower, and hence, the former require only about the same quantity of air per diem as do the latter.

The lungs are never completely emptied of air. Taking an average adult male with healthy lungs, after the most complete possible expiration his lungs will still contain about a half gallon of air. After an ordinary inspiration his lungs will contain nearly a gallon and a half, and after a very full and deep breath they will contain two gallons of air. While the difference between the possible extremes is considerable, being about one and one half gallons, the average amount of air driven out and re-inhaled at each respiration is only about one pint. The fresh air inhaled mixes with that which is already in the lungs, purifying and refreshing it, while the air which is exhaled, being heated and rarefied, ascends as colder air from below takes its place; hence, the air thrown out is not again taken back into the lungs.

Taking, for an average, eighteen respirations per minute we have 1,080 per hour, 25,920 per day, 9,468,158 per year and so on through life, and as at each breath in the adult male there is taken into the lungs, on an average, one pint, or twenty cubic inches of air, we have twelve and one-half cubic feet per hour, three hundred cubic feet per day, and 109,585 cubic feet per annum, or enough to fill a cistern sixty feet square and over thirty feet in depth.

In order that this vast quantity of air may be properly prepared for the lungs it is desirable that it should pass through the nose to be warmed, moistened and freed from dust. If the nose, through disease or defect from injury, or other cause, is not in a normal condition and cannot permit of the passage through it of the amount of air required we have a substitute—and a very poor one indeed—the habit of "mouth-breathing."

In considering the physiology of the nose we may say that its respiratory functions are as follows:

1st. To warm and rarefy the air inhaled.

2d. To moisten the same.

3d. To free the same from dust and other impurities, and

4th. By smell to detect noxious vapors.

While the external openings to the nasal passages are small, these passages rapidly increase in size as they extend backward, and owing to the convolutions of the turbinate bodies a much greater surface is obtained than could be had if these passages were shaped as is a funnel, and of no greater size than the available space would permit, and still, while the area of covering mucous membrane is quite extensive, at no point in a normal nose throughout the nasal passages proper, are two opposing surfaces further apart than is the width of the external openings, and are often much closer together. By this happy and ingenuous construction the air, as soon as it enters the nostrils, is warmed and caused to become rarefied through the absorption of heat from the rich capillary circulation of blood with which the nasal lining membrane is supplied,

The nose is divided into two parts by a vertically suspended partition which is known as the septum, the sides of which are normally plane and which, by being in the median line, divides the nose into two passages, or nostrils of equal size. While the side toward the septum of a normal nostril is smooth and even, the opposite, or outer side is very different, being composed of three shelves known as turbinates, and below these shelves three recesses known as meatuses, which jointly constitute the convolutions previously mentioned. These turbinates and meatuses, while being practically horizontal when the individual is upright, are, in fact, quite irregular in form.

The excitation of cold, or of dust, or any other irritating qualities of the air being inspired, causes a swelling of the turbinates by or through a congestion of the capillaries of the same, which capillaries are larger than those in other parts. By being so congested the increased supply of blood gives increased heat and, furthermore, through the swelling the caliber of the passages is reduced in size, causing the air to pass through under greater pressure and in a thinner column, so that the air inspired may be more thoroughly exposed to the heated turbinates. Simultaneous with the swelling of the turbinates the secretion of watery material becomes greater, so as to better attract the dust and cause its precipitation. Furthermore, evaporation of this secretion is more rapid, owing to the increased velocity of the air, the same as the strong wind dries the streets after the shower more quickly than does the gentle breeze.

The second function of the nose being to moisten the air as inspired, is secured through the evaporation of this free secretion of mucus from the lining membrane of the turbinates, which secretion is by physiologists estimated to amount to one pint daily, and which is readily evaporated by the inspired air producing the desired moisture thereof when the nasal passages are normal, but *when through deformity or disease any two opposing surfaces touch*, then at that point evaporation cannot take place and the secretion becomes thickened and often decomposed, constituting what is known as "catarrhal discharge" when blown into the handkerchief, or when discharged backward into the throat.

A second result of the touching of parts in the nasal passages is the production of a condition of inflammation of the membrane, which in time becomes chronic, and which in case of the turbinates changes the character of the secretions, rendering the evaporation thereof more difficult and increasing the tendency to catarrhal decomposition and discharge. Contact of opposing surfaces is generally produced by deformity of the septum, or else through relaxation of the turbinates, caused by their having been subjected to frequent and abrupt changes from heat to cold —a thing which in time will make these bodies relaxed and baggy. Contact causes primarily a thickening of the mucous membrane covering the turbinates which, in time, owing to pressure is often succeeded by degeneration and atrophy, particularly when the cause of contact has been an unyielding bony projection from the septum. When two surfaces in the nose are in contact, particularly when pressure exists, headache is frequently complained of.

In addition to the prevention of evaporation through contact of two opposing surfaces the presence of bony or other abnormal growths, by destroying the symmetry of the passages, may *interfere with drainage,* particularly at night when the patient is in a recumbent posture, and in this way, cause through the accumulation of secretions at some one point a thickening thereof, which in turn prevents the required evaporation, and as in case of contact may result in decomposition and catarrhal discharge.

It has been questioned how a moderate sized spur or ridge upon the septum, where no contact exists, could interfere with drainage, for the reason that while the patient is upright the projection is not at the bottom of the nasal passage. The truth of such an assertion cannot be denied, but as we spend, on an average, eight hours daily in bed it would be fair to assume that during one-third of that time, being about three hours, or one-eighth of the entire day, such spur or ridge would necessarily be at the bottom of the nostril in which it is located and would, at least for that amount of time, interfere very decidedly with drainage, much after the manner of the stone in the gutter, which during the rain when the gutter is filled with water arrests leaves, straw and other floating objects, so that after the shower, when the sun shines, other portions of the gutter are dried much sooner than the point at which the stone has served as an obstruction.

The mucous membrane covering bony projections and the convex surface of deflections of the septum will generally be found to be somewhat congested, and of a deeper color than is the membrane of the septum at other points close by. This is owing to the irritation or friction produced by the inhaled air, the projecting point being naturally struck with greater force than is the adjoining even surface. Bony projections and deviations of the septum, additionally by occluding the nasal passage, tend to induce the habit of picking the nose, and as the deviated septum, or the septum adjoining the ridge, is often of less thickness than natural, we find as a result that perforations of the septum are frequently produced.

Atrophy, or dry catarrh, is a condition generally believed to be secondary to the previously described condition of hypertrophy, and is most often found as an affection of the turbinates, though it may extend to other parts. As with hypertrophy, it may be limited to only one point, as for example, one end of one turbinate, though its tendency is progressive and if not well looked after when once begun will gradually extend to adjacent tissues. Atrophy is produced by an absorption, or degeneration, of the sub-mucous coat, which means a destruction to a greater or lesser degree of the mucous-secreting follicles, and additionally a change in the character of such secretion, whereby it is less volatile and more disposed to become inspissated. As the condition of atrophy advances the nostrils gradually increase in caliber until they become abnormally large and hence there is lost to a corresponding degree the physiological property of moistening and warming the inspired air. Such secretions as are present being too dense to evaporate become hard and incrusted, and frequently are instrumental in causing the breath to be offensive. The opening in a perforated septum by giving an abnormally large space at a local point, produces at such point the same symptoms as atrophy, even though the turbinates remain normal, for through its existence is created a space reaching from turbinate to turbinate, to the extent of the perforation, about the edges of which incrustations constantly form.

By mouth-breathing the hot air, cold, or dust, as it may chance to be, is taken directly to the lungs and is believed to often be instrumental in the development of pulmonary disease, particularly where a tendency to the same exists. Furthermore, the condition of dryness of the air causes evaporation and drying of the natural moisture of the pharynx, trachæ and bronchi which is undesirable and permits of increased friction from the passage of air through the same. The dust, which is always present in the air, is irritating to both the throat and lungs, and when profuse may block up the smaller air cells and tend to induce phthisis. The dust in the air in connection with the dryness of the pharynx, previously alluded to, is responsible for the presence of a condition of chronic sore throat. Additionally, by the breathing through the mouth of dry air the teeth are kept dry, which is harmful to them, their normal condition being one of moisture, and again, while the air is cold when inhaled it is when exhaled of blood heat, so on the ground of sudden changes of temperature we have a good reason why air should not be breathed through the mouth, for by so doing the teeth are alternately subjected, at least in winter, to temperatures tropical and frigid which is repeated twenty times a minute. Is there any wonder that in time, owing to these changes, the enamel should crack and the teeth decay ?

In addition to its physiological uses the nose also serves as a sounding-board for the voice. The so-called nasal twang, or voice, is incorrectly named, and is in fact a purely mouth voice with the nasal passages more or less occluded, giving a lack of nasal intonation. Talking with the nose closed soon tires the voice, and attempts at singing in one with stenosed nostrils produce the same effect, in proportion to the degree of stenosis, being ofttimes succeeded by hoarseness. As an illustration in proof of this statement note the effect when the nose is stopped up by a "cold in the head." The result of straining the voice is precisely the same when the tonsils are diseased or enlarged. Any abnormal condition of the nasal passages has a marked influence upon the timbre of the voice and, hence, those vocally inclined cannot build better than to have these passages made as nearly normal as possible, and the earlier in life such step is taken the better. Even and perfect teeth are also important factors in the production of voice sounds, and if the development of the teeth is influenced by nasal trouble, as is claimed, it makes the reason doubly strong why the nasal trouble should be promptly corrected.

While in anger, or during excitement the turbinates swell, showing that when under strain there is greater call upon the part of the lungs for properly prepared air. The ancient gladiator is always depicted with large nostrils and never as a mouth breather. Exercise with the nose closed would soon prove exhausting. The native Indian of by-gone days, who, as we learn from history, could stand such prolonged exertion was never a mouth breather, neither was he troubled with catarrh, for by his primitive mode of living, his freedom from stomachal troubles and from the sudden changes of temperature incidental to civilized life he escaped this most common of the compensating disadvantages of civilization.

Nasal catarrh is a disease which is very prevalent in the northern and middle portions of the United States, as well as in points further south where the air is frequently laden with dust. The sufferer from catarrh in the north will generally find his catarrh cured during a sojourn in a hot climate, which is due to a contraction of the turbinates owing to a lack of irritation, or in other words, to a diminution in the requirement of their use. In hot climates there is also not the call for as free a secretion of fluid from the turbinates, as the atmosphere is more humid than with us, resembling that of our summer sultry days.

Any deformity of the nasal passages which reduces their caliber tends to induce mouth breathing and produce nasal catarrh. Of these deformities, in recapitulation, we may particularly mention deflection, or bending of the septum and bony or cartilaginous growths thereupon; opposing the septum there may be enlargements of the turbinate bodies or else polypi attached to the turbinates. Another and very frequent cause in children of an occlusion of these passages is the presence of a sort of cauliflower growth, known as adenoid vegetations, located at the vault of the pharynx in the back part of the nose, where the nose and throat are merged one in the other. Another frequently met with trouble is a condition of enlarged tonsils which are of two varieties. The one is a condition of hypertrophy of the connective tissue—a form of tonsil known as hyperplastic—wherein the enlargement is considerable and is a kind most often found in children, and when present interferes materially in both respiration and drainage. The other is a condition more often found in adults wherein the tonsils are diseased though generally not much enlarged. This is the truly hypertrophic form, the follicles being affected while the connective tissue is not materially involved—a condition wherein the gland may be soft, though nodular at the base, and one wherein from its lacunæ is constantly being given forth an offensive, cheesy secretion. Such tonsils are quite prone to becoming inflamed from exposure to cold, and are often accompanied with cough, sore throat, post-nasal catarrh and indigestion, and merit attention owing to the effects which they produce upon the general health. They are also easily irritated by loud singing and dictating, or reading aloud, and when irritated the voice becomes husky and the throat has a sensation of rawness.

When a child has obstructions in the nasal passages, these passages and their bony framework owing to a lack of use do not grow with the rest of the body, and by not advancing with the other parts they relatively diminish in size. From this fact we may readily draw the deduction that one valuable aid in the treatment of hypertrophic catarrhal conditions, particularly with children, is the practice of *forced nasal respiration.* An examination of the back part of the mouth of a mouth-breathing child of eight or ten years of age, or older, will show the palate to be arched higher and more pointed than natural. This is directly caused by not using the nose, and through the lack of use a resulting lack of development of the bony structure thereof, which normally serves somewhat as a keystone of an arch, exerting a downward pressure, and prevents the palatine arch assuming such shape as described. The high arching of the palate causes the width of the jaws behind to be less than natural and, therefore, gives a jaw the arch of which is more pointed, and one the front teeth of which, particularly the upper ones, are often found to protrude.

While irregularities of the teeth may be produced by several causes there is no doubt but that the lack of nasal development is one of the most important factors in the causation thereof.

While a lack of nasal development originally affects the teeth of the upper jaw those of the lower jaw soon follow in the path of irregularity. Another of the objectionable effects frequently associated with the described conditions is the external lack of prominence of the nose giving the "frog-face" expression.

These are the natural train of sequences following the lack of breathing capacity of the nose, and show how disease or defect of the nose can cause disease and defect of the teeth, particularly in the way of producing irregularities. *The mouth-breathing child should at once be looked after and any deformities existing should be corrected.* The treatment of all these conditions is surgical, and thanks to the fortunate discovery of cocaine, *is comparatively painless.*

When there is present a catarrhal condition of the nasal passages, or a diseased condition of the tonsils, the inspired air is constantly being contaminated through the absorption of diseased secretions which is not infrequently manifested by the evidence of bad breath. During the process of cremation complete desiccation of the corpse is secured without its being touched by the flame, solely through the passing over and about it of heated air until all of the aqueous parts of the body are evaporated and nothing remains but a handful of ashes. It is in the same way on a smaller scale that the inspired air absorbs either the normal or diseased secretions of the nose and throat as may chance to be present, and in case the latter exist, the natural result is toward a contamination of the general health.

In mouth breathing, when the teeth are decayed, the same result is obtained and with like effect; furthermore, the secretions that have been in contact with badly decayed teeth are, when swallowed, irritating to the stomach and productive of dyspepsia, and either directly or secondarily through dyspepsia, has a tendency to produce decay in teeth not at first affected. If the secretions from a decayed tooth can, by being constantly swallowed, tend to produce dyspepsia, how much more must this be the case with diseased tonsils, the secretions from which are often offensive and in quantity far greater than from many diseased teeth. The same will apply to the swallowing of any post-nasal catarrhal discharge, or in the classic language of the advertising specialist: "catarrhal discharges which drop from the roof of the mouth."

In case of chronic pharyngitis, either with or without associated tonsilar disease, or adenoid enlargements, the condition of inflammation of the mucous membrane may extend to the gums and be observed in connection with deposits of tartar.—*Medico-Dental Bulletin.*

